# Association Between Dyspeptic Symptoms and *Helicobacter pylori* Stool Antigen Positivity: A Retrospective Study

**DOI:** 10.3390/reports9030230

**Published:** 2026-07-19

**Authors:** Maryam Izadi, Amir Mirnateghi, Shiva Shafabakhsh

**Affiliations:** 1 Chiropractic Department, Keiser University, West Palm Beach, FL 33411, USA; 2 College of Osteopathic Medicine, LECOM University, Bradenton, FL 34211, USA; 3 Walgreens, West Palm Beach, FL 33441, USA

**Keywords:** *Helicobacter pylori*, stool antigen test, epigastric pain, dyspepsia, gastrointestinal symptoms, retrospective study

## Abstract

**Background/Objectives**: *Helicobacter pylori* infection is a major cause of chronic gastritis, peptic ulcer disease, and gastric cancer. Although accurate diagnostic tests are available, their cost, accessibility, and invasiveness may limit their routine use, particularly in resource-limited settings. Because dyspeptic symptoms are frequently used to guide testing decisions, identifying symptom patterns associated with *H. pylori* infection may improve patient selection for diagnostic testing. This study evaluated the association between gastrointestinal symptoms, particularly burning epigastric pain that worsens on an empty stomach, and *H. pylori* stool antigen positivity. **Methods**: This retrospective observational study included 589 adults who underwent *H. pylori* stool antigen testing at a private laboratory in Tehran, Iran, between May 2021 and June 2022. Patients were classified as *H. pylori*-positive (*n* = 353) or *H. pylori*-negative (*n* = 236) based on stool antigen test results. Gastrointestinal symptoms documented in patient records were compared between groups using chi-square analysis. The sensitivity, specificity, positive predictive value (PPV), and negative predictive value (NPV) of burning epigastric pain were also calculated. **Results**: Burning epigastric pain that worsens on an empty stomach was significantly more common in *H. pylori*-positive than *H. pylori*-negative patients (76.2% vs. 4.2%; *p* < 0.00001). Significant associations were also observed for bloating, persistent vomiting, dysphagia, diarrhea, constipation, and melena. Burning epigastric pain demonstrated a sensitivity of 76.2%, specificity of 95.8%, PPV of 96.4%, and NPV of 72.9%. Overall, 99.7% of *H. pylori*-positive patients reported at least one gastrointestinal symptom compared with 35.2% of *H. pylori*-negative patients. **Conclusions**: Burning epigastric pain that worsens on an empty stomach was strongly associated with *H. pylori* stool antigen positivity and may help clinicians identify patients who are more likely to benefit from diagnostic testing. However, symptoms alone are insufficient for diagnosis and should complement, rather than replace, established diagnostic methods. Prospective studies using standardized symptom assessment and multiple diagnostic modalities are needed to validate these findings.

## 1. Introduction

*Helicobacter pylori* (*H. pylori*) is a Gram-negative, spiral-shaped bacterium that colonizes the gastric mucosa and infects more than half of the world’s population [1]. Infection is more common in regions with poor sanitation, household overcrowding, and lower socioeconomic conditions and remains a major global public health problem [2,3,4,5]. Chronic *H. pylori* infection is a well-established cause of chronic gastritis and peptic ulcer disease and is an important risk factor for gastric adenocarcinoma and mucosa-associated lymphoid tissue (MALT) lymphoma [3,6,7,8,9]. Because eradication therapy can reduce disease-related complications, early diagnosis and treatment are essential.

Several invasive and noninvasive methods are available for diagnosing *H. pylori* infection. Invasive techniques include endoscopy with histology, rapid urease testing, culture, and molecular assays, whereas noninvasive approaches include the urea breath test, stool antigen test (SAT), and serology [10,11,12,13,14,15,16,17]. Among these, the urea breath test and stool antigen test are widely recommended for detecting active infection because of their high diagnostic accuracy [14,15,16,17,18,19]. Nevertheless, access to these tests may be limited by cost, availability, and healthcare resources, particularly in low- and middle-income countries, creating a need for strategies to improve patient selection for diagnostic testing.

Although dyspeptic symptoms have long been associated with *Helicobacter pylori* infection, the predictive value of individual symptoms remains controversial because dyspepsia encompasses a heterogeneous group of nonspecific gastrointestinal complaints [20,21,22,23]. Most previous studies have evaluated dyspepsia as a broad clinical syndrome rather than examining the association of individual symptoms with active *H. pylori* infection confirmed by stool antigen testing [8,20,21,22,23]. In addition, relatively few studies have specifically investigated burning epigastric pain that worsens on an empty stomach while simultaneously examining age-specific symptom patterns within a large referral-based clinical population.

Therefore, the present retrospective observational study aimed to evaluate the association between individual gastrointestinal symptoms and *H. pylori* stool antigen positivity in adults undergoing routine clinical testing. Particular emphasis was placed on burning epigastric pain that worsens on an empty stomach, exploratory measures describing the association between symptoms and stool antigen positivity, and age-stratified symptom patterns. Rather than establishing new diagnostic criteria, this study seeks to provide additional real-world evidence on symptom patterns to help clinicians identify patients more likely to benefit from confirmatory *H. pylori* testing, particularly in healthcare settings with limited diagnostic resources.

## 2. Materials and Methods

### 2.1. Study Design and Setting

This single-center retrospective observational study was conducted using patient records from Wilson Laboratory (Shush Square, Tehran, Iran). Medical records of patients who underwent *Helicobacter pylori* stool antigen testing between 22 May 2021 and 11 June 2022 were reviewed to evaluate the association between gastrointestinal symptoms and *H. pylori* infection.

### 2.2. Study Population Eligible Patients

A total of 719 patient records were screened. Patients were eligible if they were ≥18 years of age and had undergone *H. pylori* stool antigen testing with complete demographic and clinical information. Records with missing demographic or clinical data were excluded (*n* = 130). Additional exclusion criteria included pregnancy, malignancy, liver, renal, or hematologic disease, recent use (within four weeks before testing) of proton pump inhibitors, H_2_-receptor antagonists, bismuth compounds, antibiotics (including amoxicillin, clarithromycin, or metronidazole), or nonsteroidal anti-inflammatory drugs, as well as documented allergy to these medications. Records with incomplete demographic information, missing documentation of gastrointestinal symptoms, indeterminate stool antigen test results, or insufficient clinical information were excluded before analysis. Data extraction was performed using predefined eligibility criteria to minimize errors and improve consistency during retrospective medical record review. After applying the eligibility criteria, 589 patients were included in the final analysis.

The minimum required sample size was estimated using a standard sample-size formula, with a calculated minimum of 185 participants. The final sample exceeded this requirement.

### 2.3. Data Collection

Patient records were reviewed for age, sex, medical history, medication history, presenting gastrointestinal symptoms, and *H. pylori* stool antigen test results. Patients were classified as *H. pylori*-positive or *H. pylori*-negative based on stool antigen test results.

The gastrointestinal symptoms evaluated included bloating, nausea, persistent vomiting, burning epigastric pain that worsens when the stomach is empty, dysphagia, unintentional weight loss (>3 kg during the preceding month), early satiety or loss of appetite, diarrhea, constipation, and melena.

### 2.4. Stool Antigen Testing

Active *H. pylori* infection was determined using a commercially available stool antigen enzyme immunoassay performed as part of routine clinical practice at the laboratory. Because of the retrospective nature of the study, detailed information regarding the assay manufacturer, antibody type (monoclonal or polyclonal), and antigen targets was unavailable.

### 2.5. Statistical Analysis

Categorical variables are presented as frequencies and percentages. Associations between gastrointestinal symptoms and *H. pylori* infection were evaluated using the chi-square test. Statistical significance was defined as a two-sided *p*-value < 0.05.

The diagnostic performance of burning epigastric pain that worsens on an empty stomach was summarized by calculating sensitivity, specificity, positive predictive value (PPV), and negative predictive value (NPV) using stool antigen test results as the reference classification. Descriptive analyses were then performed across the predefined age groups (18–25, 26–33, 34–41, 42–49, 50–57, 58–65, and ≥65 years) to evaluate symptom prevalence throughout adulthood. Descriptive analyses were also performed across predefined age groups (18–25, 26–33, 34–41, 42–49, 50–57, 58–65, and ≥65 years) to evaluate symptom prevalence throughout adulthood.

### 2.6. Ethical Approval

This study was reviewed and determined to be exempt by the Keiser University Institutional Review Board under Category 4 of 45 CFR 46.104 because it involved retrospective analysis of existing de-identified patient records. The exemption was granted on 5 December 2025. Individual informed consent was waived because all data were de-identified before analysis.

### 2.7. Data Availability

The data analyzed in this study are not publicly available because they contain information derived from patient medical records. De-identified data may be made available by the corresponding author upon reasonable request, subject to institutional and ethical requirements.

## 3. Results

### 3.1. Study Population

A total of 719 patient records were screened for eligibility. After excluding 130 records that did not meet the inclusion criteria, 589 patients were included in the final analysis. Based on stool antigen test (SAT) results, 353 patients (59.9%) were classified as *Helicobacter pylori*-positive and 236 (40.1%) as *H. pylori*-negative.

Overall, 352 of 353 (99.7%) *H. pylori*-positive patients reported at least one gastrointestinal symptom compared with 83 of 236 (35.2%) *H. pylori*-negative patients (*p* < 0.00001) (Table 1).

### 3.2. Association Between Gastrointestinal Symptoms and H. pylori Infection

Burning epigastric pain that worsens when the stomach is empty was the symptom most strongly associated with *H. pylori* positivity, occurring in 269 of 353 (76.2%) infected patients compared with 10 of 236 (4.2%) non-infected patients (*p* < 0.00001).

Other symptoms that were significantly more frequent among *H. pylori*-positive patients included bloating (*p* = 0.012), persistent vomiting (*p* = 0.029), dysphagia (*p* < 0.00001), diarrhea (*p* = 0.0003), constipation (*p* = 0.0001), and melena (*p* < 0.00001). No statistically significant associations were observed for nausea (*p* = 0.093), unintentional weight loss (*p* = 0.100), or early satiety/loss of appetite (*p* = 0.791) (Table 1).

### 3.3. Exploratory Symptom Performance Measures

Burning epigastric pain that worsens when the stomach is empty demonstrated a sensitivity of 76.2%, specificity of 95.8%, positive predictive value (PPV) of 96.4%, and negative predictive value (NPV) of 72.9% for identifying *H. pylori* stool antigen positivity (Table 2). The estimated odds ratio indicated a strong association between this symptom and *H. pylori* infection.

### 3.4. Symptom Distribution Across Age Groups

Age-stratified analyses demonstrated that burning epigastric pain remained significantly associated with *H. pylori* positivity across all age groups examined (*p* < 0.00001 for each group). The prevalence of this symptom among infected patients ranged from 68.6% to 87.9%, whereas it remained uncommon among *H. pylori*-negative patients.

**Figure 1:**
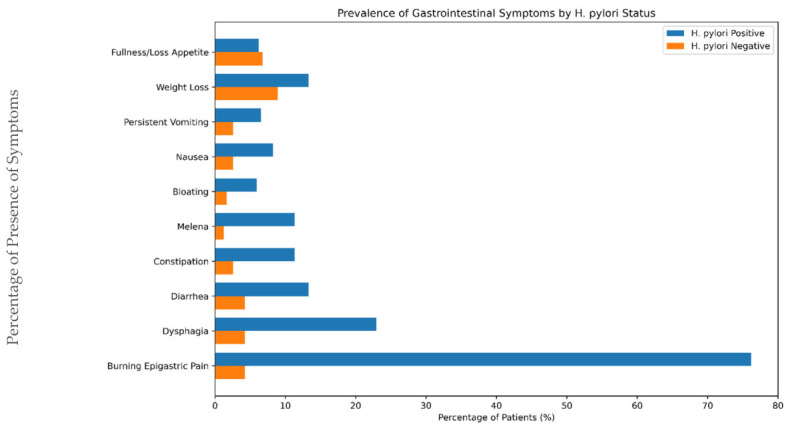
The Prevalence of *H. pylori*’s Most Common Symptoms Across All Age Groups.

**Figure 2:**
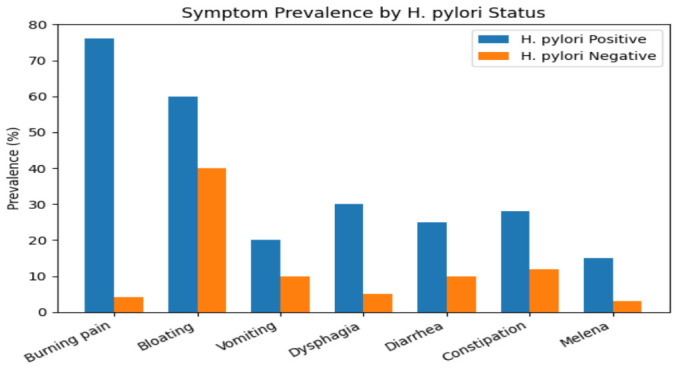
Prevalence of gastrointestinal symptoms in *H. pylori*–positive and negative patients.

Several additional symptoms, including dysphagia, diarrhea, bloating, constipation, and melena, showed significant associations in specific age groups; however, these findings were not consistent across all age categories. In contrast, burning epigastric pain consistently demonstrated the strongest association with *H. pylori* positivity regardless of age (Table 2, Table 3, Table 4, Table 5, Table 6, Table 7, Table 8 and Table 9; Figure 1 and Figure 2).

Distribution of the Most Common H. pylori-Related Symptoms Among Different Age Groups

The strength of the associations varied across age groups. In this retrospective cohort, burning epigastric pain that worsens on an empty stomach had a sensitivity of 76.2%, specificity of 95.8%, positive predictive value of 96.4%, and negative predictive value of 72.9% for *Helicobacter pylori* stool antigen positivity (Table 2). These exploratory performance measures describe the association observed within this referral-based study population and should not be interpreted as definitive estimates of diagnostic accuracy.

Odds Ratio (OR) Calculation

Burning Pain That Worsens on an Empty Stomach

Using the combined cohort data:

- *H. pylori*–positive patients with the symptom: 269
- *H. pylori*–positive patients without symptoms: 84
- *H. pylori*–negative patients with the symptom: 10
- *H. pylori*–negative patients without symptoms: 226
- Positive: 269/353 → a = 269, b = 353 − 269 = 84
- Negative: 10/236 → c = 10, d = 236 − 10 = 226

$$OR=\frac{269\cdot 226}{84\cdot 10}=\frac{60794}{840}\approx 72.4$$

The odds ratio (OR) for worsening of epigastric burning epigastric pain that worsens on an empty stomach was approximately 72.4, indicating a strong association with *H. pylori* stool antigen positivity in this retrospective cohort.

However, this estimate should be interpreted cautiously because the study was retrospective and referral-based, and the symptom was uncommon among *H. pylori*–negative patients. Therefore, the OR reflects an observed association within this selected population rather than a generalizable risk estimate or evidence of causation.

### 3.5. Symptom Patterns Across Age Groups

Age-stratified analysis demonstrated that the prevalence and statistical significance of individual symptoms varied between age groups. Despite this variation, burning epigastric pain that worsens on an empty stomach remained the most consistently associated symptom among *H. pylori*–positive patients.

Symptoms such as dysphagia, diarrhea, constipation, and melena reached statistical significance in selected age groups but were not uniformly observed across the entire cohort. In contrast, symptoms including nausea, persistent vomiting, bloating, and loss of appetite generally showed weaker or inconsistent associations with *H. pylori* positivity (Figure 2).

The proportion of symptomatic patients was consistently higher in the *H. pylori*–positive group than in the *H. pylori*–negative group across all age categories.

### 3.6. Prevalence of H. pylori–Associated Symptoms Across Age Groups (Table 4, Table 5, Table 6, Table 7, Table 8 and Table 9)

#### 3.6.1. Age Group 18–25 Years (Table 4)

In the 18–25-year age group, 67 patients were included, of whom 33 tested positive for *H. pylori* and 34 tested negative. No significant difference in infection rates was observed between males and females (*p* = 0.067).

In this age group, burning epigastric pain that worsens on an empty stomach (*p* < 0.00001) and melena (black, tarry stools) (*p* = 0.018) were significantly associated with infection. The remaining symptoms, including bloating (*p* = 1.00), nausea (*p* = 0.57), persistent vomiting (*p* = 0.54), dysphagia (*p* = 0.06), unintentional weight loss ≥3 kg/month (*p* = 0.96), early satiety or loss of appetite (*p* = 0.08), diarrhea (*p* = 0.96), and constipation (*p* = 0.08), did not reach statistical significance.

#### 3.6.2. Age Group 26–33 Years (Table 5)

Of the 97 patients aged 26–33 years, 57 were *H. pylori*–positive and 40 were *H. pylori*–negative. No statistically significant difference in infection prevalence between males and females was observed (*p* = 0.67).

Burning epigastric pain that worsens on an empty stomach (*p* < 0.00001), dysphagia (*p* = 0.002), and melena (black, tarry stools) (*p* = 0.03) were significantly associated with *H. pylori* infection in this age group. Diarrhea showed a borderline association (*p* = 0.054). Other symptoms, including bloating (*p* = 0.77), nausea (*p* = 0.50), persistent vomiting (*p* = 0.20), unintentional weight loss ≥3 kg within one month (*p* = 0.55), early satiety or loss of appetite (*p* = 0.36), and constipation (*p* = 0.21), were not statistically significant.

#### 3.6.3. Age Group 34–41 (Table 6)

Among 125 patients, 70 tested positive, and 55 tested negative. No significant difference was found between males and females (*p* = 0.17). “Burning pain that worsens when the stomach is empty” (*p* < 0.00001), dysphagia (*p* = 0.029), diarrhea (*p* = 0.015), unintentional weight loss >3 kg/month (*p* = 0.009), and bloody/black tarry stools (*p* = 0.003) were significantly associated with infection. Other symptoms, including bloating (*p* = 0.44), nausea (*p* = 0.50), persistent vomiting (*p* = 0.27), feeling of fullness/loss of appetite (*p* = 0.24), and constipation (*p* = 0.17), were not significant.

#### 3.6.4. Age Group 42–49 (Table 7)

Of 87 patients, 56 tested positive, and 31 tested negative. A significant gender difference was observed (*p* = 0.018). Only “burning pain that worsens when the stomach is empty” (*p* < 0.00001) was statistically significant. Nausea (*p* = 0.0589) and bloody/black, tarry stools (*p* = 0.0589) were more common among patients with positive results but did not reach statistical significance. Other symptoms, including bloating (*p* = 0.19), persistent vomiting (*p* = 0.29), dysphagia (*p* = 0.10), unintentional weight loss >3 kg/month (*p* = 0.82), feeling of fullness/loss of appetite (*p* = 0.24), diarrhea (*p* = 0.10), and constipation (*p* = 0.64), were not significant.

#### 3.6.5. Age Group 50–57 (Table 8)

Among 104 patients, 66 tested positive, and 38 tested negative. No significant gender difference was found (*p* = 0.46). Significant symptoms included bloating (*p* = 0.025), burning pain that worsens when the stomach is empty (*p* < 0.00001), dysphagia (*p* = 0.006), and diarrhea (*p* = 0.025). Other symptoms, such as nausea (*p* = 0.72), persistent vomiting (*p* = 0.91), unintentional weight loss >3 kg/month (*p* = 0.61), feeling of fullness/loss of appetite (*p* = 0.48), constipation (*p* = 0.10), and bloody/black tarry stools (*p* = 0.12), were not significant.

#### 3.6.6. Age Group 58–65 (Table 9)

Among 55 patients, 33 tested positive and 22 tested negative for *H. pylori*. No significant gender difference was observed (*p* = 0.54). Only “burning pain that worsens when the stomach is empty” (*p* < 0.00001) was significantly associated with *H. pylori* positivity. Other symptoms, including bloating (*p* = 0.22), nausea (*p* = 0.52), persistent vomiting (*p* = 0.52), dysphagia (*p* = 0.22), unintentional weight loss >3 kg/month (*p* = 0.78), feeling of fullness/loss of appetite (*p* = 0.77), diarrhea (*p* = 0.52), constipation (*p* = 0.52), and bloody/black tarry stools (*p* = 0.72), were not statistically significant.

#### 3.6.7. Summary of Main Findings (Table 10)

Among all evaluated symptoms, burning epigastric pain that worsens when the stomach is empty showed the strongest association with *H. pylori* stool antigen positivity in this laboratory-based cohort. However, symptom prevalence and statistical significance varied across age groups, and several symptoms demonstrated overlapping distributions between positive and negative patients.

**Table 10:** The Prevalence of *H. pylori*’s Most Common Symptoms Across All Age Groups.

Age Groups/Presented Symptoms	18–25	26–33	34–41	42–49	50–57	58–65	>65 (66–83)	Combined (N= 589)	
	Positive	Positive	Positive	Positive	Positive	Positive	Positive	Positive	Neg
**Bloating**	0.00%	3.51%	4.29%	5.36%	12.12% *	0.00%	13.16%	5.95% *	1.69%
**Nausea**	3.03%	5.26%	8.57%	10.71%	6.06%	9.09%	15.79%	8.22%	4.66%
**Persistent Vomiting**	6.06%	0.00%	5.71%	3.57%	3.03%	9.09%	13.16%	6.52% *	2.54%
**Burning Pain That Worsens When the Stomach Is Empty**	87.88% *	75.44% *	68.57% *	75.00% *	87.88% *	72.73% *	65.79% *	76.20% *	4.24%
**Dysphagia**	21.21%	29.82% *	18.57% *	14.29%	22.73% *	15.15%	42.11% *	22.95% *	4.24%
**Unintentional Weight Loss (Over 3 kg in Past Month)**	12.12%	14.04%	11.43% *	14.29%	7.58%	21.21%	18.42%	13.31%	8.90%
**Feeling of Fullness/Loss of Appetite**	0.00%	1.75%	11.43%	3.57%	4.55%	3.03%	18.42%	6.23%	6.78%
**Diarrhea**	15.15%	8.77% *	14.29% *	14.29%	12.12% *	9.09%	21.05%	13.31% *	4.24%
**Constipation**	15.15%	8.77%	7.14%	12.50%	12.12%	9.09%	18.42%	11.33% *	2.54%
**Bloody or Black Tarry Stools**	15.15% *	10.53% *	14.29% *	10.71%	6.06%	12.12%	13.16%	11.33% *	1.27%
**Total Number of Patients Presented with Symptoms**	96.97% *	100.00% *	100.00% *	100.00% *	100.00% *	100.00% *	100.00% *	99.72% *	35.17%

* Indicates that there is a statistically significant association between the prevalence of the symptom and *H. pylori* positive test result.

## 4. Discussion

Previous studies have reported associations between dyspeptic symptoms and *Helicobacter pylori* infection, although most have evaluated dyspepsia as a broad clinical syndrome rather than examining the contribution of individual symptoms [2120–2423]. Because dyspepsia encompasses a heterogeneous group of nonspecific gastrointestinal complaints that overlap with many other gastrointestinal disorders, its value in predicting *H. pylori* infection has remained controversial [2120–2423]. In addition, relatively few studies have specifically investigated burning epigastric pain that worsens on an empty stomach in relation to stool antigen-confirmed infection while simultaneously exploring age-specific symptom patterns within a large laboratory referral population. Rather than identifying a previously unrecognized symptom, the present study extends existing evidence by demonstrating the consistency of this symptom’s association across adult age groups and by providing exploratory estimates of symptom performance in a real-world clinical setting.

The exploratory diagnostic performance measures observed in this study further support this association. Burning epigastric pain that worsens on an empty stomach demonstrated high specificity and positive predictive value relative to stool antigen positivity, whereas sensitivity and negative predictive value were more moderate. The extremely large odds ratio observed for burning epigastric pain primarily reflects the rarity of this symptom among stool antigen-negative patients in this selected referral cohort rather than a causal effect or a universally applicable risk estimate. These findings suggest that the presence of this symptom may increase clinical suspicion of *H. pylori* infection; however, its absence does not reliably exclude infection. Sensitivity, specificity, positive predictive value (PPV), and negative predictive value (NPV) were included solely to describe the observed symptom distribution within this retrospective cohort and should not be interpreted as validation of symptom-based diagnosis.

Several additional gastrointestinal symptoms, including dysphagia, diarrhea, constipation, melena, bloating, and persistent vomiting, were also more common among *H. pylori*-positive patients. However, these associations varied across age groups and were less consistent than those observed for burning epigastric pain. While previous studies have examined dyspepsia broadly, relatively few have focused specifically on burning epigastric pain that worsens on an empty stomach or evaluated its consistency across multiple adult age groups using stool antigen-confirmed infection. The present study therefore extends existing evidence by providing age-stratified analyses and exploratory estimates of symptom performance within a real-world laboratory referral population. Although several symptom associations varied across age groups, burning epigastric pain remained consistently associated with *H. pylori* positivity. Because these subgroup analyses were exploratory and not powered for formal comparisons between age groups, these findings should be interpreted with caution.

The findings should be interpreted in light of several limitations. First, the retrospective study design limited control over data quality and the completeness of symptom documentation. Although patients with recent use of proton pump inhibitors, H_2_-receptor antagonists, antibiotics, bismuth compounds, and other medications known to interfere with stool antigen testing were excluded according to the predefined eligibility criteria, undetected false-negative results cannot be completely excluded in retrospective studies. Consequently, some degree of outcome misclassification remains possible.

Second, the study population consisted exclusively of patients referred for stool antigen testing at a single private laboratory, introducing the possibility of referral and selection bias. Because only patients referred for stool antigen testing were included, referral bias likely enriched the cohort for symptomatic individuals. Consequently, the prevalence of symptoms and the strength of the observed associations may differ substantially from those in unselected primary care or general population settings, thereby limiting the generalizability of these findings.

Third, *H. pylori* infection was classified using stool antigen testing without confirmation by an independent reference standard such as the urea breath test or biopsy-based methods. The stool antigen assay was used as the clinical reference test because this information was available from routine laboratory records. The absence of confirmation by urea breath testing or endoscopic biopsy may have resulted in some misclassification of outcomes. Therefore, the reported odds ratios and exploratory diagnostic performance measures should be interpreted as descriptive findings within this selected cohort rather than validated estimates of diagnostic accuracy. The extremely large odds ratio observed for burning epigastric pain primarily reflects the rarity of this symptom among stool antigen-negative patients in this selected referral cohort rather than a causal effect or a universally applicable risk estimate.

In addition, multivariable regression analyses were not performed; therefore, potential confounding by age, sex, coexisting gastrointestinal disorders, socioeconomic characteristics, medication use, and overlapping gastrointestinal symptoms could not be evaluated. Because the available retrospective medical records lacked complete and standardized information on several clinically relevant confounders, multivariable adjustment was not considered appropriate. Because several gastrointestinal symptoms frequently coexisted in individual patients, the study cannot determine whether individual symptoms are independent predictors of infection or simply reflect a greater overall symptom burden among *H. pylori*-positive patients. Furthermore, because multiple unadjusted comparisons were performed, the possibility of type I error cannot be excluded, and statistically significant findings should be interpreted as exploratory. Accordingly, these findings should be interpreted with caution until confirmed in prospective studies using multivariable analyses.

An additional strength of this study is that it reflects routine clinical practice by analyzing consecutive patients referred to for stool antigen testing rather than highly selected research participants. Although this referral-based design limits generalizability, it provides insight into symptom patterns encountered in everyday diagnostic practice.

Despite these limitations, this study has several strengths. It included a relatively large cohort of patients evaluated in routine clinical practice and systematically examined a broad spectrum of gastrointestinal symptoms across multiple adult age groups. Rather than attempting to validate symptom-based diagnoses, the study provides clinically relevant observational evidence on symptom patterns that may help clinicians identify patients more likely to benefit from confirmatory *H. pylori* testing, particularly in healthcare settings with limited diagnostic resources.

Future prospective, multicenter studies incorporating standardized symptom assessment, multivariable statistical analyses, adjustment for potential confounders, and comparison with established reference diagnostic methods are needed to validate these findings and further clarify the clinical value of symptom-based approaches for selecting patients for *H. pylori* testing.

## 5. Conclusions

This retrospective observational study identified a significant association between burning epigastric pain that worsens on an empty stomach and *Helicobacter pylori* stool antigen positivity in a selected clinical population undergoing evaluation for dyspeptic symptoms. Several additional gastrointestinal symptoms were also more common among *H. pylori*-positive patients, although these associations were less consistent across age groups.

Rather than establishing new diagnostic criteria, this study provides additional evidence supporting the clinical relevance of burning epigastric pain that worsens on an empty stomach as one component of symptom assessment when selecting patients for confirmatory *H. pylori* testing. These findings may provide additional clinical information to guide patient selection for confirmatory *H. pylori* testing, particularly in healthcare settings with limited diagnostic resources. However, because this was a retrospective observational study conducted in a referral-based population, the findings should be considered exploratory and require confirmation in prospective, multicenter studies using standardized symptom assessment, multivariable analyses, and independent reference diagnostic methods.

## Figures and Tables

**Table 1:** Combined Results of the Prevalence of Symptoms in All Patients Based on Negative and Positive *H. pylori* Lab Results.

Symptom	*H. pylori* Negative n/N (%)	*H. pylori* Positive n/N (%)	*p*-Value
Bloating	4/236 (1.7)	21/353 (6.0)	0.012
Nausea	6/236 (2.5)	29/353 (8.2)	0.006
Persistent vomiting	6/236 (2.5)	23/353 (6.5)	0.029
Burning epigastric pain worsening on an empty stomach	10/236 (4.2)	269/353 (76.2)	<0.00001 *
Dysphagia	10/236 (4.2)	81/353 (23.0)	<0.00001 *
Weight loss (>3 kg/month)	21/236 (8.9)	47/353 (13.3)	0.103
Early satiety/loss of appetite	16/236 (6.8)	22/353 (6.2)	0.791
Diarrhea	10/236 (4.2)	47/353 (13.3)	0.0004 *
Constipation	6/236 (2.5)	40/353 (11.3)	0.0003 *
Melena (black tarry stools)	3/236 (1.3)	40/353 (11.3)	0.00001 *
Any reported symptom	83/236 (35.2)	352/353 (99.7)	<0.00001 *

* Symptoms with Significantly Higher Prevalence Among H. pylori-Positive Patients.

**Table 2:** summarizes the diagnostic performance for burning epigastric pain.

Metric	Value
**Sensitivity**	76.2%
**Specificity**	95.8%
**Positive Predictive Value (PPV)**	96.4%
**Negative Predictive Value (NPV)**	72.9%

**Table 3:** Other Symptoms (ORs Approximate).

Symptom	a	b	c	d	OR
**Dysphagia**	81	272	10	226	6.7
**Diarrhea**	47	306	10	226	3.5
**Constipation**	40	313	6	230	4.9
**Bloody/Black Stools**	40	313	3	233	9.97

**Table 4:** The Prevalence of Symptoms in Patients Based on Negative and Positive *H. pylori* Lab Results (Ages 18–25).

	Tested Positive for *H. pylori*			Tested Negative for *H. pylori*			
Age Group 18–25	Total Number	Symptomatic Patients	% of Symptomatic Patients	Total Number	Symptomatic Patients	% of Symptomatic Patients	*p* Value
**Gender**	14 (F) 19 (M)			22 (F) 12 (M)			0.06744
**Bloating**	33	0	0.00%	34	0	0.00%	1.00000
**Nausea**	33	1	3.03%	34	2	5.88%	0.57252
**Persistent Vomiting**	33	2	6.06%	34	1	2.94%	0.53707
**Burning Pain That Worsens When the Stomach Is Empty**	33	29	87.88%	34	0	0.00%	0.00001 *
**Dysphagia**	33	7	21.21%	34	2	5.88%	0.06582
**Unintentional Weight Loss (Over 3 kg in Past Month)**	33	4	12.12%	34	4	11.76%	0.96411
**Feeling of Fullness/Loss of Appetite**	33	0	0.00%	34	3	8.82%	0.08082
**Diarrhea**	33	5	15.15%	34	5	14.71%	0.95918
**Constipation**	33	5	15.15%	34	1	2.94%	0.08013
**Bloody or Black Tarry Stools**	33	5	15.15%	34	0	0.00%	0.01830
**Total Number of Patients Presented with Symptoms**	33	32	96.97%	34	16	47.06%	0.00001 *

* Symptoms with Significantly Higher Prevalence Among H. pylori-Positive Patients.

**Table 5:** The Prevalence of Symptoms in Patients Based on Negative and Positive *H. pylori* Lab Results (Ages 26–33).

	Tested Positive for *H. pylori*			Tested Negative for *H. pylori*			
Age Group 26–33	Total Number	Symptomatic Patients	% of Symptomatic Patients	Total Number	Symptomatic Patients	% of Symptomatic Patients	*p* Value
**Gender**	31 (F) 26 (M)			20 (F) 20 (M)			0.67022
**Bloating**	57	2	3.51%	40	1	2.50%	0.77756
**Nausea**	57	3	5.26%	40	1	2.50%	0.50048
**Persistent Vomiting**	57	0	0.00%	40	1	2.50%	0.23016
**Burning Pain That Worsens When the Stomach Is Empty**	57	43	75.44%	40	3	7.50%	0.00001 *
**Dysphagia**	57	17	29.82%	40	2	5.00%	0.00242
**Unintentional Weight Loss (Over 3 kg in Past Month)**	57	8	14.04%	40	4	10.00%	0.55240
**Feeling of Fullness/Loss of Appetite**	57	1	1.75%	40	2	5.00%	0.36339
**Diarrhea**	57	5	8.77%	40	0	0.00%	0.05443
**Constipation**	57	5	8.77%	40	1	2.50%	0.20685
**Bloody or Black Tarry Stools**	57	6	10.53%	40	0	0.00%	0.03413
**Total Number of Patients Presented with Symptoms**	57	57	100.00%	40	13	32.50%	0.00001 *

* Symptoms with Significantly Higher Prevalence Among H. pylori-Positive Patients.

**Table 6:** The Prevalence of Symptoms in Patients Based on Negative and Positive *H. Pylori* Lab Results (Ages 34–41).

		Tested Positive for *H. pylori*		Tested Negative for *H. pylori*			
Age Group 34–41	Total Number	Symptomatic Patients	% of Symptomatic Patients	Total Number	Symptomatic Patients	% of Symptomatic Patients	*p* Value
**Gender**	43 (F) 27 (M)			27 (F) 28 (M)			0.16777
**Bloating**	70	3	4.29%	55	1	1.82%	0.43652
**Nausea**	70	6	8.57%	55	3	5.45%	0.50337
**Persistent Vomiting**	70	4	5.71%	55	1	1.82%	0.26985
**Burning Pain That Worsens When the Stomach Is Empty**	70	48	68.57%	55	3	5.45%	0.00001 *
**Dysphagia**	70	13	18.57%	55	3	5.45%	0.02934
**Unintentional Weight Loss (Over 3 kg in Past Month)**	70	8	11.43%	55	0	0.00%	0.00956
**Feeling of Fullness/Loss of Appetite**	70	8	11.43%	55	3	5.45%	0.24187
**Diarrhea**	70	10	14.29%	55	1	1.82%	0.01459
**Constipation**	70	5	7.14%	55	1	1.82%	0.16685
**Bloody or Black Tarry Stools**	70	10	14.29%	55	0	0.00%	0.00347
**Total Number of Patients Presented with Symptoms**	70	70	100.00%	55	15	27.27%	0.00001 *

* Symptoms with Significantly Higher Prevalence Among H. pylori-Positive Patients.

**Table 7:** The Prevalence of Symptoms in Patients Based on Negative and Positive *H. pylori* Lab Results (Ages 42–49).

	Tested Positive for *H. pylori*			Tested Negative for *H. pylori*			
Age Group 42–49	Total Number	Symptomatic Patients	% of Symptomatic Patients	Total Number	Symptomatic Patients	% of Symptomatic Patients	*p* Value
**Gender**	27 (F) 29 (M)			23 (F) 8 (M)			0.01891
**Bloating**	56	3	5.36%	31	0	0.00%	0.18969
**Nausea**	56	6	10.71%	31	0	0.00%	0.05892
**Persistent Vomiting**	56	2	3.57%	31	0	0.00%	0.28710
**Burning Pain That** **Worsen When Stomach Is Empty**	56	42	75.00%	31	2	6.45%	0.00001 *
**Dysphagia**	56	8	14.29%	31	1	3.23%	0.10475
**Unintentional Weight Loss (Over 3 kg in the Past** **Month)**	56	8	14.29%	31	5	16.13%	0.81734
**Feeling of Fullness/Loss of Appetite**	56	2	3.57%	31	3	9.68%	0.24123
**Diarrhea**	56	8	14.29%	31	1	3.23%	0.10475
**Constipation**	56	7	12.50%	31	5	16.13%	0.63827
**Bloody or Black Tarry Stools**	56	6	10.71%	31	0	0.00%	0.05892
**Total Number of Patients Presented with Symptoms**	56	56	100.00%	31	11	35.48%	0.00001 *

* Symptoms with Significantly Higher Prevalence Among H. pylori-Positive Patients.

**Table 8:** The Prevalence of Symptoms in Patients Based on Negative and Positive *H. pylori* Lab Results (Ages 50–57).

	Tested Positive for *H. pylori*			Tested Negative for *H. pylori*			
Age Group 50–57	Total Number	Symptomatic Patients	% of Symptomatic Patients	Total Number	Symptomatic Patients	% of Symptomatic Patients	*p* Value
**Gender**	48 (F) 18 (M)			25 (F) 13 (M)			0.45637
**Bloating**	66	8	12.12%	38	0	0.00%	0.02550
**Nausea**	66	4	6.06%	38	3	7.89%	0.71924
**Persistent Vomiting**	66	2	3.03%	38	1	2.63%	0.90687
**Burning Pain That Worsens When the Stomach Is Empty**	66	58	87.88%	38	0	0.00%	0.00001 *
**Dysphagia**	66	15	22.73%	38	1	2.63%	0.00624
**Unintentional Weight Loss (Over 3 kg in Past Month)**	66	5	7.58%	38	4	10.53%	0.60631
**Feeling of Fullness/Loss of Appetite**	66	3	4.55%	38	3	7.89%	0.48055
**Diarrhea**	66	8	12.12%	38	0	0.00%	0.02550
**Constipation**	66	8	12.12%	38	1	2.63%	0.09742
**Bloody or Black Tarry Stools**	66	4	6.06%	38	0	0.00%	0.12171
**Total Number of Patients Presented with Symptoms**	66	66	100.00%	38	13	34.21%	0.00001 *

* Symptoms with Significantly Higher Prevalence Among H. pylori-Positive Patients.

**Table 9:** The Prevalence of Symptoms in Patients Based on Negative and Positive *H. pylori* Lab Results (Ages 58–65).

	Tested Positive for *H. pylori*			Tested Negative for *H. pylori*			
Age Group 58–65	Total Number	Symptomatic Patients	% of Symptomatic Patients	Total Number	Symptomatic Patients	% of Symptomatic Patients	*p* Value
**Gender**	25 (F) 8 (M)			15 (F) 7 (M)			0.53656
**Bloating**	33	0	0.00%	22	1	4.55%	0.21645
**Nausea**	33	3	9.09%	22	1	4.55%	0.52482
**Persistent Vomiting**	33	3	9.09%	22	1	4.55%	0.52482
**Burning Pain That Worsens When Stomach Is Empty**	33	24	72.73%	22	0	0.00%	0.00001 *
**Dysphagia**	33	5	15.15%	22	1	4.55%	0.21645
**Unintentional Weight Loss (Over 3 kg in Past Month)**	33	7	21.21%	22	4	18.18%	0.78313
**Feeling of Fullness/Loss of Appetite**	33	1	3.03%	22	1	4.55%	0.76870
**Diarrhea**	33	3	9.09%	22	1	4.55%	0.52482
**Constipation**	33	3	9.09%	22	1	4.55%	0.52482
**Bloody or Black Tarry Stools**	33	4	12.12%	22	2	9.09%	0.72397
**Total Number of Patients Presented with Symptoms**	33	33	100.00%	22	9	40.91%	0.00001 *

* Symptoms with Significantly Higher Prevalence Among H. pylori-Positive Patients.

## Data Availability

The original data presented in this study are available on reasonable request from the corresponding author. The data are not publicly available due to privacy concerns.
